# An Alarming Case of Hemorrhage, from the Extraction of Two Molar Teeth

**Published:** 1859-11

**Authors:** W. H. Shadoan


### AN ALARMING CASE OF HEMORRHAGE, FROM THE EXTRACTION OF TWO MOLAR TEETH.

BY W. H. SHADOAN.

Miss E. L., aged 17 years, of scrofulous diathesis, called, on Monday, August 28th, to have some teeth extracted. On examining her teeth, I found them badly decayed. She said she wished several removed if she could endure the operation. I commenced by removing the first inferior molar of the right side. She did not complain much, and concluded to have another extracted. Then took out the antagonizing tooth of the upper jaw. She concluded not to have any more extracted at that time, and well enough she did not, as it turned out. She went home a short distance in the country, about half a mile. The hemorrhage continued until Wednesday, when it entirely ceased, but on Thursday about noon, the hemorrhage broke out from the upper socket, and bled so alarmingly that the family became frightened, and sent for the family physician; he being rather unwell, and not thinking her in danger, did not go, but sent her some tannin, told them how to use it, and insisted that they should send for me. They did not send for me, however, but let it run on until 7 o'clock the next morning, when the young lady came to me herself. I examined the parts, and found that the blood was emitted from the posterior-palatin edge of the socket. I first tried tannin, then tannin and morphia, mixed with tincture of myrrh, which seemed to stop the blood for a short time. I then took of tannin about ten grains, of sulphate of morphine about ten grains, dissolved with oil of cloves; took a small portion of cotton, and saturated well with the mixture, and placed it in the orifice. In a few moments the hemorrhage had entirely stopped.

She in the mean time had lost so much blood that she was getting quite weak. She started home, and on the way the bleeding commenced from the inferior socket, and in a few hours she was reduced so much that she could hardly sit up

About half-past 1 o'clock she came in again, and it was with difficulty that I got the hemorrhage stopped at all. I began to think that I was going to have trouble with it. I tried the remedies as before, at first, to no avail, but at last succeeded in getting it checked, in the evening, with tannin, creosote, morphine, and opium.

Sulphate of copper is very good in such cases, but I did not use it in the case above referred to.

